# From CRISPR screens to circuits: identifying key regulators in T cell activation and state transitions

**DOI:** 10.1038/s41392-025-02200-3

**Published:** 2025-04-07

**Authors:** Maria Silvia Roman Azcona, Toni Cathomen, Claudio Mussolino

**Affiliations:** 1 Institute for Transfusion Medicine and Gene Therapy, Medical Center - University of Freiburg, Freiburg, Germany; 2 Center for Chronic Immunodeficiency (CCI), Medical Center - University of Freiburg, Freiburg, Germany; 3 Faculty of Medicine, University of Freiburg, Freiburg, Germany

**Keywords:** Lymphocytes, Adaptive immunity

A recent study published in *Nature* by Maya M. Arce and colleagues unveils the role of central gene circuits in governing the delicate balance between T cell rest and T cell activation.^1^ This work bridges fundamental molecular biology with findings in preclinical models, providing insights into context-specific gene regulation and potential therapeutic targets for immune modulation.

The immune system relies on highly specialized cells to maintain homeostasis and respond to external threats. Among these, the CD4^+^ T cell compartment exhibits remarkable versatility, including T cell subsets with almost antagonistic functions, and that are capable of toggling between resting (quiescent) and activated states depending on environmental cues. The IL-2 receptor alpha subunit (IL-2Rα or CD25) serves as a critical marker for T cell activation, with distinct expression profiles in regulatory T cells (Treg) and effector T cells (Teff), characterized by either its constitutive or transient upregulation upon stimulation, respectively. Despite its importance, the trans-regulatory networks orchestrating these state-specific responses remain incompletely understood. Therefore, screening interventions that modulate IL-2Rα expression can serve as a means of identifying genes involved in T cell activation, offering valuable insights by comparing the regulatory mechanisms between Teff and Treg cells.

**Fig. 1:**
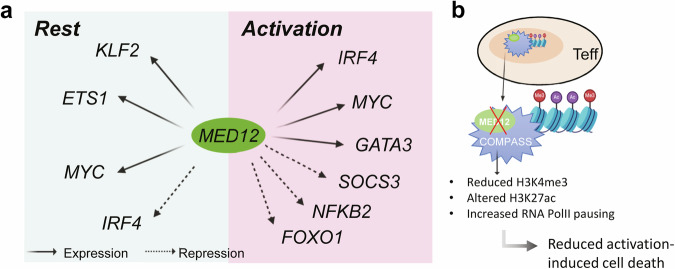
Role of MED12 in CD4^+^ T cells. **a** MED12 is a powerful regulator during CD4^+^ T cell rest and activation. Its expression correlates with activation and repression of different genes, depending by the T cell state. For effector T cells, MED12 was found to negatively affect *IRF4* expression while inducing *KLF2*, *ETS1* and *MYC* in the resting state. In contrast, during activation MED12 can promote the activation of *IRF4*, *MYC* and *GATA3* genes while contributing to dampen the expression of *SOCS3*, *NFKB2* and FOXO1. This complex interaction highlights the critical role of MED12 as an orchestrator of T cells rest and activation. **b** MED12-ablated T effector cells showed altered H3K27ac levels and decreased H3K4me3 at some key IL-2Rα regulators and globally increased pausing of RNA Pol II upon stimulation which eventually results in reduced activation-induced cell death. This suggests that its mechanism of action involves diverse epigenetic changes as a result of its participation in the Mediator complex and/or by interacting with the histone-methylating complex COMPASS. (Created with BioRender.com)

The primary objective of this study was to identify and characterize the regulators of IL-2Rα expression across different T cell states. To this end, the authors conducted a series of CRISPR screens in primary human Treg and Teff cells to uncover context-dependent regulators and explore their role in maintaining resting and activated states. Using a designed library of 6,000 sgRNAs targeting over a thousand genes coding for transcription factors and chromatin modifiers in resting and stimulated Teff and Treg, they mapped dynamic gene circuits that highlighted context-specific effects, with only a small subset of regulators acting uniformly across conditions. The study identified the Mediator complex subunit 12 (MED12) as a key orchestrator with opposing roles: a negative regulator of IL-2Rα in resting Teff but a positive regulator in stimulated Teff and Treg. Notably, MED12 regulates the expression of both rest-maintaining and activation-promoting factors, such as KLF2 and MYC, respectively, establishing it as a central player in T cell state transitions capable of either promoting resting stability or facilitating activation transitions (Fig. 1).

Subsequent experiments confirmed MED12’s role in modulating IL-2Rα expression and broader T cell gene expression networks. The authors used Perturb-CITE-seq, which allowed them to converge data from CRISPRi perturbations with single-cell transcriptomics and proteomics, providing enough data to assign activation scores resulting from each of the perturbations. This is particularly important, because although IL-2Rα is a canonical marker of Teff activation, its mere expression does not guarantee a correlation with T cell activation.^2^ Interestingly, targeted knockout experiments showed altered activation features, thus confirming the critical role of MED12 in both reaching a fully activated state and returning to a full resting state.

MED12 is part of the kinase module of the Mediator complex, a multiprotein complex pivotal for regulating gene transcription by communicating transcription factors and RNA polymerase II, thus, facilitating the transcription of nearly all RNA polymerase II-dependent genes. To investigate the mechanism by which MED12 exerts its function in the context of T cell activation, the authors performed a thorough analysis of the MED12 interactome. Immunoprecipitation mass spectrometry showed that MED12 interacts with members of the histone-methylating complex COMPASS, suggesting a role in H3K4 methylation. Chromatin immunoprecipitation (ChIP) and CUT&RUN analyses confirmed MED12’s interactions with critical epigenome modifying factors, underscoring its role in regulating histone modifications at key loci, including *IL2RA* and *KLF2* at rest or *MYC* and *SATB1* after stimulation. While these findings highlight a critical role for MED12 in regulating state-specific gene expression, the lack of enzymatic activity suggests a more complex mechanism to coordinate the action of the complex Mediator function that remains to be understood.^3^

This work emphasizes that the resting state must be actively maintained by multiple interrelated factors, of which KLF2 appears to be the most relevant, and that activation-promoting factors must repress them to allow activation signals. Although the active nature of the resting state has been proposed previously, the identification of the key players in this mechanism will be critical to better understand how this seemingly “static state” is maintained. Providing detailed maps of the regulatory circuits controlling T cell states, as in this study, will certainly have significant implications for immunotherapy. Indeed, recent work has shown that ablation of MED12 in CAR T cells is beneficial for tumor control, but the precise mechanism was unclear.^4^ This research unveiled that MED12 knockout could improve cell persistence by limiting activation-induced cell death, and, by manipulating MED12 and other regulators, it outlines a framework for designing next-generation CAR T cells with enhanced durability, resistance to apoptosis, and improved adaptability to the tumor microenvironment, paving the way for more effective and resilient CAR T cell therapies in clinical settings.

The study from Alexander Marson’s lab sheds important new light on how T cell states are regulated, but some caveats warrant further investigation. First, ex vivo cultured human T cells may not fully capture the in vivo complexity of immune responses, accentuating the need for better models. Second, the existence of compensatory pathways or redundant genes may have contributed to the oversight of other critical effectors. Additionally, the role of MED12 in other immune cell types or under chronic stimulation conditions remains unexplored. For example, it would be interesting to see if these results can be extended to CD8^+^ T cells, given that they share certain characteristics but differ profoundly from CD4^+^ T cells in their overall phenotype and function.^5^ Finally, the long-term consequences of manipulating MED12-mediated networks, particularly with respect to immune tolerance and memory, remain to be determined. Future studies will extend these findings to broader immune contexts and assess viable therapeutic approaches that may emerge from such investigations.

Arce et al. provide transformative insights into the molecular control of T cell states. By elucidating the dynamic interplay of gene circuits and identifying MED12 as a central regulator, this work advances our understanding of immune regulation and paves the way for innovative therapeutic interventions.
